# STAT3-Dependent Gene TRIM5γ Interacts With HBx Through a Zinc Binding Site on the BBox Domain

**DOI:** 10.3389/fmicb.2021.663534

**Published:** 2021-07-02

**Authors:** Hongxiao Song, Fengchao Xu, Xiaoli Pang, Qingfei Xiao, Qi Wei, Bingxin Lei, Xiaolu Li, Xixi Fan, Guangyun Tan

**Affiliations:** ^1^Department of Immunology, Institute of Translational Medicine, The First Hospital of Jilin University, Changchun, China; ^2^Department of Pediatric Gastroenterology, The First Hospital of Jilin University, Changchun, China; ^3^Department of Nephrology, The First Hospital of Jilin University, Changchun, China; ^4^Department of Anesthesia, The First Hospital of Jilin University, Changchun, China; ^5^Department of Clinical Specialty of Integrated Traditional Chinese and Western Medicine, Changchun University of Chinese Medicine, Changchun, China

**Keywords:** STAT3, HBx, TRIM5, interferon, HBV – hepatitis B virus

## Abstract

Owing to its broad-spectrum antivirus activities, interferon (IFN) is an important alternative agent for use in the treatment of hepatitis B virus (HBV)-infected patients; however, the mechanism involved in the inhibition of HBV infection and replication by IFN remains unclear. We previously reported that the induction of TRIM5γ is important in the IFN treatment of HBV patients as it promotes the degradation of the HBx protein, while the manner in which TRIM5γ is induced by IFN and how TRIM5γ interacts with HBx remain unestablished until date. Our present findings confirmed the TRIM5γ-HBx-DDB1 interactions in the HBV-infected Primary human hepatocytes (PHH), and we further found that STAT3, and not STAT1, was responsible for the induction of TRIM5γ upon IFN stimulation and that the zinc binding site His123 on the BBOX domain was a decisive site in the interaction between TRIM5γ BBOX and HBx. In addition, based on the BBOX domain, we detected a 7-amino acid peptide with the potential of promoting HBx degradation and inhibiting HBV replication. On the other hand, we noted that the TRIM5γ expression was inhibited by HBV in chronically HBV infected patients. Thus, our study identified the crucial role of STAT3 in the induction of TRIM5γ, as well as proposed a 7-amino acid, small peptide as a potential candidate for the development of therapeutic agents targeting HBx.

## Introduction

In response to most of the virus infection, interferon (IFN) is immediately induced and approximately 300 IFN-stimulated gene expression is further induced toward the inhibition of virus replication (Liu et al., 2012). Unfortunately, it has been reported that the IFN signaling is inhibited in HBV-infected patients (Cheng et al., 2017). Interferon therapy is an approved treatment modality for chronic HBV infection (Jacobson, 2006). However, the poor response and the substantial side-effects associated with this treatment affect its clinical utility. In addition, besides nucleoside/nucleotide analog, there is currently no much effective treatment available for patients who respond poorly to IFN therapy (Tan et al., 2018a). Recently, IFNa14 was reported as the most effective IFN subtype in the inhibition of HBV cccDNA transcription and HBeAg/HBsAg production (Chen et al., 2021); however, HBV persistence remains a huge issue that requires resolution (Nassal, 2015).

A total of 10 different genotypes of HBV (A–J) have been reported from all across the world (Sunbul, 2014). HBV is a partially double-stranded DNA virus whose genome is located in the nucleus of infected hepatocytes (Nassal, 2015). All HBV viral RNAs are transcripted from the covalently closed circular DNA (cccDNA) that acts as the transcription template (Levrero et al., 2009). Presently, cccDNA application has failed to eradicate infected hepatocytes by using the available HBV therapeutics (such as IFN-α and antiviral drugs), despite it inhibits new viral DNA replication (Shih et al., 2016). Consequently, persistent cccDNA in the nucleus of hepatocytes leads to post-treatment viral rebound (Revill and Locarnini, 2016).

The TRIM family members are involved in multiple cellular signaling, especially in exerting antiviral activities (Ozato et al., 2018). In addition, TRIM E3 ubiquitin ligases have been found to restrict viral replication by directly targeting the viral-related proteins (Rajsbaum et al., 2014). HBx, one of the proteins expressed by the HBV genome, interacts with several host factors and plays an indispensable role in the HBV replication and infection (Kim et al., 2016; Decorsiere et al., 2016; Xu et al., 2019). In our previous study, we noted that TRIM5γ promoted HBx degradation and that the BBox domain of TRIM5γ was sufficient to ubiquitinate HBx (Tan et al., 2019). In the present study, we therefore went further to evaluate how BBox interacts with HBx and what is the mechanism involved in the induction of TRIM5γ after IFN stimulation.

## Materials and Methods

### Samples

In this study, we enrolled 10 healthy individuals and 20 HBV-infected patients (Supplementary Table 1), and their serum and peripheral blood mononuclear cells (PBMCs) were collected from the venous blood samples. The protocol of this prospective clinical sampling study was approved by the institutional review board of the First Hospital, Jilin University.

### Cell Culture, Plasmids, and Reagents

HepG2 and HEK293T cells were maintained in Dulbecco’s Modified Eagle’s Medium supplemented with 10% inactivated fetal bovine serum, including penicillin (100 IU/mL) and streptomycin (100 mg/mL) under a 5% CO_2_ atmosphere at 37°C. PHH cells were pruchased from RILD (Research Institute for liver Diseases, Shanghai, China), HBx antibody (GS968942, Gilead Sciences) was kindly provided as a gift from Dr. Simon Fletcher. The expression constructs of TRIM5γ were generated by cloning the sequence of the coding region into a VR1012 expression vector. Anti-GAPDH antibody, anti-HA-tag, and GST-tag antibody were obtained from Proteintech. Human IFN-α was purchased from Peprotech (Jiangsu, China). DDB1, STAT1, and STAT3 antibodies were procured from Cell Signaling Technology (Danvers, MA, United States). Anti-tubulin antibody was obtained from Santa Cruz Biotechnology (Santa Cruz, CA, United States), Antibodies against TRIM5γ and Smc6 were purchased from Abcam (Shanghai, China), Stattic was obtained from Sigma (Danvers, MA, United States).

### PHH Infection and HBx Pull Down Assay

The supernatants of HepG2.2.15 cells, which were derived from the human hepatoma cell line HepG2 transfected with the full genome of HBV, were collected and concentrated. PHH cells were seeded into collagen-coated 60-mm plates and cultured overnight. Cells were inoculated for 24 h with a multiplicity of infection (MOI) of 500 genome equivalents (Geq) per cell; after infection, cells were washed three times with PBS and were maintained in maintenance medium containing 2% DMSO and 2% FBS, and the cells were then incubated at the incubator. 4 days later, cells were collected and whole cell lysates were made and subjected to pull down assay, briefly, for immunoprecipitation, dynabeads protein G (Selleck) were incubated with 10 μg of monoclonal rabbit anti-HBx antibody (GS968942, Gilead Sciences), DDB1 (#5428, CST) or IgG for 10 min at room temperature. 2 mg of protein lysate was added to the dynabeads antibody complex and rotated overnight at 4°C. The beads were washed for 5 times, and boiled with 30 ul protein loading buffer and subjected to immunoblotting on the next day.

### RNA Extraction and Quantitative Real-Time PCR (q-RT-PCR)

Total RNA was extracted from the cells by using the EasyPure RNA Kit (Transgen, China) according to the manufacturer’s instructions and then converted to first-strand cDNA using the TransScript First-Strand cDNA Synthesis SuperMix (Transgen). HBV DNA was isolated from supernatants as per the manufacturer’s instructions (Transgen). A housekeeping gene, GAPDH, was used as an internal control for quantitation, and the gene expression was quantified as previously described. The gene-specific primer sequences used for qRT-PCR are shown in Supplementary Table 2.

### Co-immunoprecipitation and Western Blotting

Between 24 and 48 h after the transfection of the expression plasmids, the cells were lysed with 50 mM Tris–HCl (pH 8.0), 150 mM NaCl, and 1% NP-40 containing cocktail inhibitors (Millipore). The cell lysates were immunoprecipitated and then incubated with the ANTI-FLAG^®^ M2 Affinity Gel (Sigma) for overnight. Immunoblotting was performed as previously described (Tan et al., 2018c). Briefly, the cells were collected and lysed in ice-cold cell lysis buffer for 30 min, with tapping of the tubes performed every 10 min. The protein concentration was quantified by the Coomassie Plus^TM^ Protein Assay Reagent (Thermo Scientific). The band intensities were quantified with the ChemiDoc^TM^ XRS + Molecular Imager software (Bio-Rad). The samples were separated by SDS-PAGE and transferred onto polyvinylidene difluoride membranes. The blots were blocked in Tris-buffered saline containing 0.1% Tween-20 and 5% skimmed milk and then probed with the relevant antibodies.

### Enzyme-Linked Immunosorbent Assay

The HepG2 cells were mock-transfected or transfected with TRIM5γ-expression plasmids together with the pHBV1.3-HBV expression plasmids. The supernatant was collected after 72 h and then subjected to ELISA to detect the levels of HBV e-antigen (HBeAg) and HBV surface antigen (HBsAg) (Kehua Biotech, China).

### CRISPR/Cas9 Knockout

Plasmids expressing Cas9 and sgRNA were co-transfected into HepG2 cells using lip2000 transfection reagent (Invitrogen). At 36-h post-transfection, the cells were selected with puromycin at 2 μg/mL. After 2 days, the living cells were added to a 96-well plate at a cell density of 1 cell/well. Immunoblotting was performed with TRIM5γ-specific antibodies to ensure the gene knockout (KO) results and DNA sequencing was performed to further confirm the results of gene knockout. The sequences of the sgRNAs are listed in Supplementary Table 2.

### TRIM5 Promoter Reporters and Dual-Luciferase Reporter Assay

The TRIM5γ promoter reporters were generated by cloning the promoter region sequence into a pGL4.1 expression vector. The HepG2 cells were transfected with the respective TRIM5γ promoter reporters together with a pGL4.74 Tk-Rluc reporter. After 16 h, the cells were treated with IFN-α. After treatment, the cells were lysed with a passive lysis buffer and the activities of firefly luciferase and Renilla luciferase in the lysates were measured with the Dual-Luciferase Reporter Assay System (Promega).

### Statistical Analysis

The results were presented as mean ± SD and analyzed using the Student’s *t*-test. *P* < 0.05 was considered indicative of statistically significant differences.

## Results

### STAT3 Plays a Critical Role in the TRIM5γ Induction Upon IFN Stimulation

**FIGURE 1:**
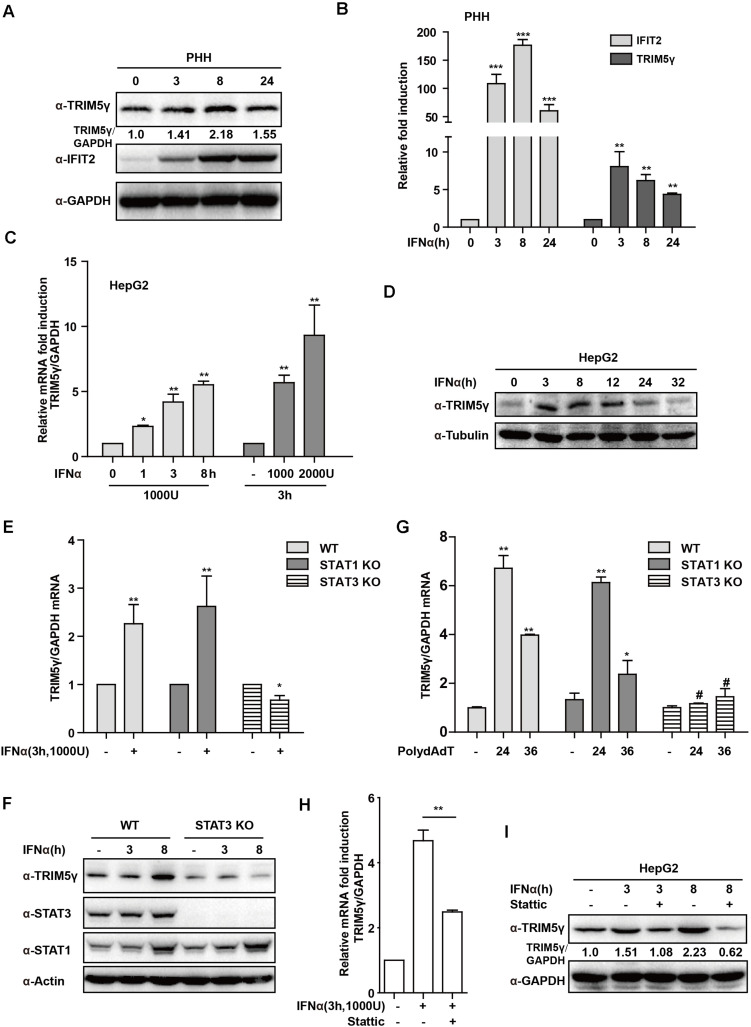
IFN-induced TRIM5γ in a STAT3-dependent manner. **(A–D)** PHH or HepG2 cells were treated with IFN-α, as indicated, and the whole-cell lysates were immunoblotted with TRIM5γ and GAPDH antibodies. RNA was extracted and qRT-PCR was conducted to determine the TRIM5γ expression. **(E–G)**. HepG2 WT or STAT1 or 3 KO cells were treated with IFN-α or transfected with polydAdT, as indicated. TRIM5γ expression was determined by qRT-PCR or immunoblotting. **(H,I)**. The HepG2 cells were treated with IFNα without or with Stattic (10 μM), as indicated. The TRIM5γ expression was determined by western blotting and q-PCR. The data are presented as mean ± SD from 3 independent experiments. The Student’s *t*-test was applied to analyze the results. *p < 0.05; **p < 0.01; ^#^*p* > 0.05.

In our recent study, we reported that TRIM5γ inhibited HBV replication (Tan et al., 2019). To further investigate the mechanism of TRIM5γ induction by IFN in the present study, we first stimulated PHH or HepG2 cells with IFN-α in a dose- and time-dependent manner. As expected, the TRIM5γ induction as confirmed by both q-PCR and western blotting, IFIT2, a classical Interferon Simulated Gene, was used as a positive control (Figures 1A–D). STAT1 was the major response transcriptional factor in the type-I IFN signaling pathway. Thus, we questioned whether STAT1 is critical in the induction of TRIM5γ; however, TRIM5γ remained upregulated in the STAT1 knockout cells, which was the same cell line we used previously (Tan et al., 2018b). Interestingly, the induction was blocked in the STAT3 knockout cells (Figures 1E,F), which we used as the control cells. To confirm this finding, PolydAdT, which was used as a DNA virus stimulation and induced huge amounts of interferon, was transfected into the WT or STAT1 or 3 KO cells, and as expected, TRIM5γ induction was noted in STAT1 but not in STAT3 KO cells (Figure 1G). In addition, the STAT3 inhibitor Stattic was used to treat the HepG2 cells together with IFN and the induction was inhibited (Figures 1H,I). Collectively, all these data suggested that STAT3, and not STAT1, was the major factor in the IFN-induced-TRIM5γ upregulation.

### STAT3 Binds to the TRIM5γ Promoter

**FIGURE 2:**
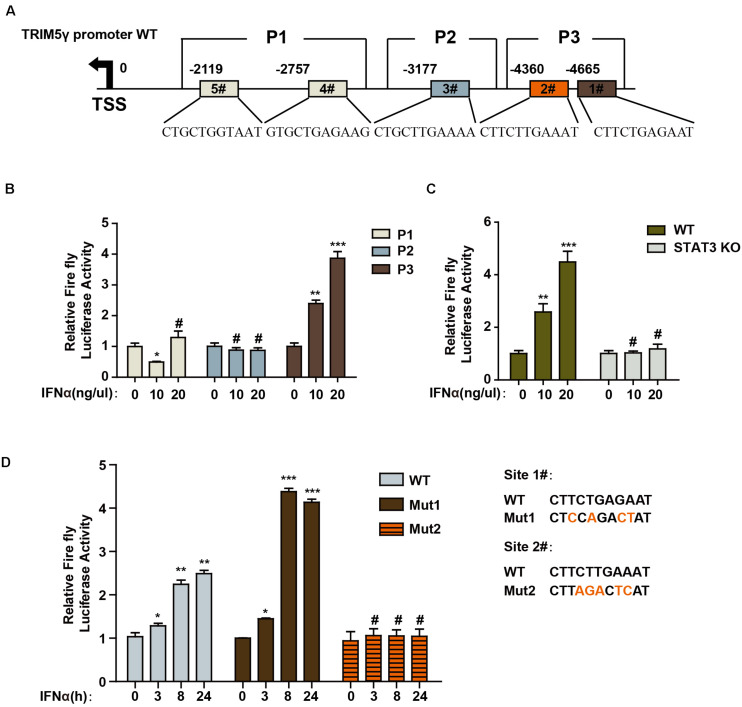
STAT3 promotes TRIM5γ transcription by binding to the promoter. **(A)** Schematic illustration showing potential STAT3 binding sites in the TRIM5γ promoter region. **(B)** HepG2 cells were transfected with luciferase reporters harboring wild-type (WT) promoter regions of TRIM5γ together with pGL4.74 TK-Luc reporter; After 16 h, the cells were treated with IFN-α (10 ng/mL); the luciferase activity was measured 24 h after the treatment. Firefly luciferase activity was first normalized to that of Renilla luciferase, and fold induction of the relative luciferase activity over the control group was plotted as shown. **(C)** WT or STAT3 KO HepG2 cells were treated or transfected, as indicated, and then analyzed as shown in **B**. **(D)** HepG2 cells were transfected with luciferase reporters harboring WT or Mut promoter regions of TRIM5γ together with pGL4.74 TK-Luc reporter and treated as indicated, and analyzed as shown in **B**. Data are presented as mean ± SD from 3 independent experiments. The Student’s *t*-test was applied to analyze the results. *p < 0.05; **p < 0.01; ***p < 0.001; ^#^*p* > 0.05.

To further determine the mechanism of TRIM5γ induction in response to IFN-α stimulation, we predicted the potential STAT3-binding sites in the human TRIM5γ promoter region (5000 bps) by JASPAR (Mathelier et al., 2016). Three regions of the promoter (P1, P2, and P3), which included 1 or 2 predicted STAT3-binding sites, were inserted into the pGL4.1 luciferase reporter construct (Figure 2A). We found that IFN-α stimulation significantly induced the P3-luc activity (Figure 2B); however, this induction was blocked in the STAT3 Knockout cells (Figure 2C). This event indicated that STAT3 is the activator of TRIM5γ promoter luciferase activity. In order to further confirm the STAT3-binding site, we mutated the potential sites individually in the P3 region (Figure 2D). Interestingly, only Site #2 mutation blocked the luciferase activity induced by IFN treatment. Collectively, all these data indicated that STAT3 may directly bind to the promoter of TRIM5γ and induced its transcription.

### HBx Binds to the C-terminal of TRIM5γ BBox Domain

**FIGURE 3:**
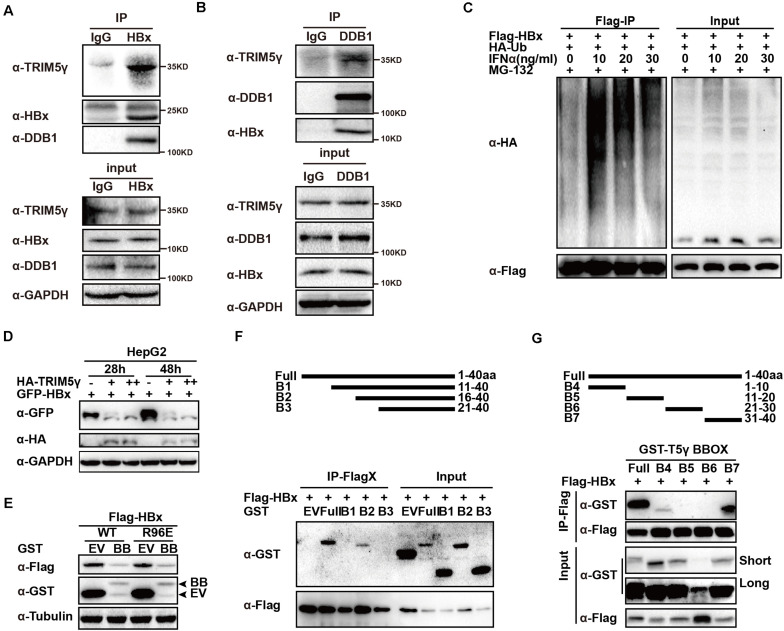
HBx binds to the C-terminal of TRIM5γ BBox domain. **(A,B)** PHH cells were infected with HBV and subjected to HBx or DDB1 pull down assay, the whole-cell lysates were immunoblotted with TRIM5γ, DDB1, HBx, and GAPDH antibodies. **(C)** HepG2 cells were transfected with Flag-HBx and HA-Ub plasmids, as indicated, 8 h before the collection, and the cells were treated by MG132 (10 uM) and the whole-cell lysates were immunoblotted with HA and Flag antibodies. **(D)** HepG2 WT cells were transfected with GFP-HBx and HA-TRIM5γ plasmids, as indicated, and the whole-cell lysates were immunoblotted with HA, GFP, and GAPDH antibodies. **(E)** HepG2 cells were co-transfected with BBox and HBx WT or the R96E expression plasmids, as indicated, 36 h later, the whole cell lysates were immunoblotted with Flag, GST, or Tubulin antibodies. **(F,G)** 293T cells were transfected with Flag-HBx plasmids or co-transfected with BBox peptides expression plasmids, as indicated, and subjected to Co-IP analysis. The cell lysates and precipitated samples were subjected to immunoblotting using the indicated antibodies.

In this study, we confirmed the TRIM5γ-HBx-DDB1 interactions in the HBV-infected PHH cells by HBx or DDB1 pulldown assay (Figures 3A,B and Supplementary Figure 1), and in addition to our research on TRIM5γ BBox domain promoting HBx degradation, we wanted to determine the binding sites of TRIM5γ by HBx. IFN promoted HBx ubiquitination and degradation by inducing TRIM5γ expression (Figures 3C,D). The BBox domain of TRIM5γ was found to be sufficient to trigger HBx (WT or R96E, an HBx mutation blocks the interaction of HBx-DDB1-Smc complexes) degradation (Figure 3E). In order to further investigate how this small peptide interacted with HBx, truncated BBox constructs were generated and CoIP was performed by co-transfection with an HBx construct. Our results indicated that HBx interacted with B2 truncation. However, both B1 and B3 protein was not stable in the cells and degraded into small fragments, therefore the interaction was not detected (Figure 3F and Supplementary Figure 2). In addition, after we truncated BBox into 4 peptides, we found that the interaction region was at the end of the BBox domain (shown as B7 in Figure 3G). Thus, these data cumulatively indicated that HBx bind to the C-terminal region of BBox.

### H123 in the BBox Domain Is the Binding Site of HBx-BBox Interaction

**FIGURE 4:**
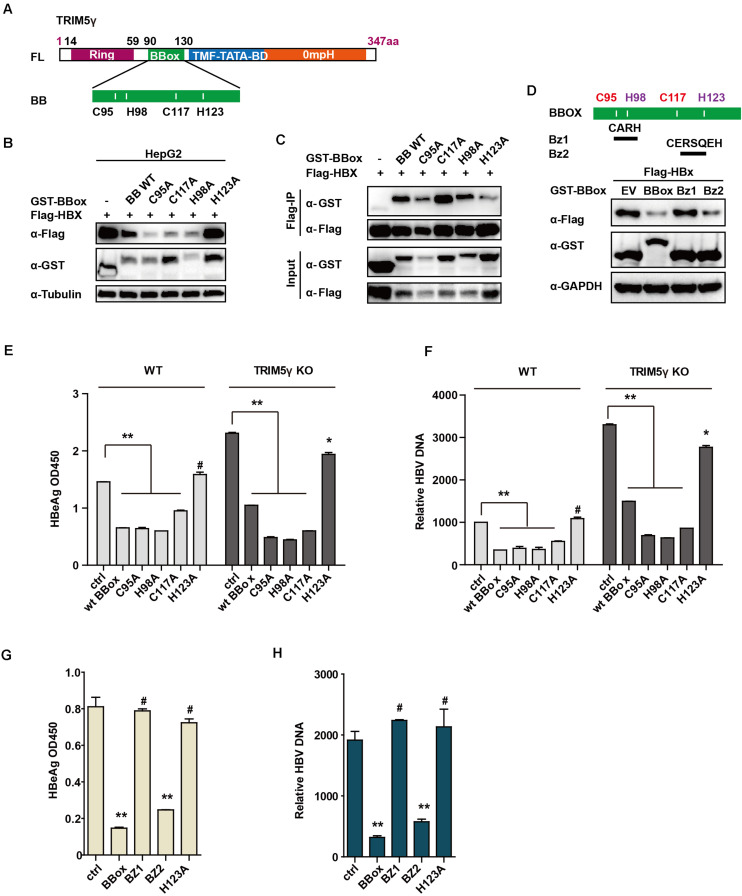
H123 is critical in the HBx-BBox interaction. **(A)** Schematic illustration showing mutants of TRIM5γ BBox. **(B)**, HepG2 were transfected with BBox WT or mutant plasmids, as indicated. The cell lysates and precipitated samples were subjected to immunoblotting using the indicated antibodies. **(C)**, 293T cells were transfected with Flag-HBx plasmids or co-transfected with BBox mutants, as indicated, and then subjected to Co-IP analysis. **(D)** HepG2 cells were transfected with BBox WT, BZ1, or BZ2 plasmids, along with HBx plasmids as indicated. The cell lysates and precipitated samples were subjected to immunoblotting using the indicated antibodies. **(E,F)** HepG2 WT or TRIM5 KO cells were transfected with pHBV1.3 plasmids or co-transfected with BBox WT or mutants, and after 48 h, HBeAg and HBV DNA were analyzed by ELISA or Q-PCR. Data are presented as mean ± SD from three independent experiments. The Student’s *t*-test was applied to analyze the results. **(G,H)** HepG2 cells were transfected with pHBV1.3 plasmids or co-transfected with BBox WT, BZ1, BZ2, or H123 mutant, and after 48 h, HBeAg and HBV DNA were analyzed by ELISA or Q-PCR. Data are presented as mean ± SD from three independent experiments. The Student’s *t*-test was applied to analyze the results. *p < 0.05; **p < 0.01; ^#^*p* > 0.05.

To identify the critical site on the BBox domain that interacts with HBx, individual plasmids encoding mutated zinc binding sites of the BBox domain was generated. Interestingly, the BBox-induced degradation of HBx was rescued after H123 site mutation, but not the others in HepG2 cells. Moreover, we further confirmed that the interaction between HBx and BBox was almost blocked after H123 mutation (Figures 4A–C). Small peptides are believed to act as good candidates for drug development (Crook et al., 2020). We wondered whether a much smaller peptide can promote HBx degradation and accordingly generated constructs encoding two peptides (as indicated in Figure 4D). Surprisingly, HBx was degraded by BZ2, but not BZ1 (Figure 4D), which was consistent with our results that H123 is critical in HBx degradation. We further investigated the function of H123 in the regulation of the HBV replication. HepG2 TRIM5 KO cells were generated by the CRISPR/Cas9 technology, and WT or mutants of BBox was co-transfected with pHBV1.3 plasmids into the HepG2 WT and KO cells. As expected, HBV replication was significantly inhibited in WT BBox and mutants, including C95A, H98A, C117A plasmids transfected HepG2 cells, but not that significant in H123A mutant-transfected cells (Figures 4E,F), suggesting that H123 in the BBox domain was indispensable in the interaction and degradation of HBx as well as in the inhibition of HBV replication. In addition, on investigating the role of BZ1 and BZ2 in restricting HBV replication, we found that BZ2 was consistent with our expectation (Figures 4G,H).

### HBV Inhibits TRIM5γ Expression

**FIGURE 5:**
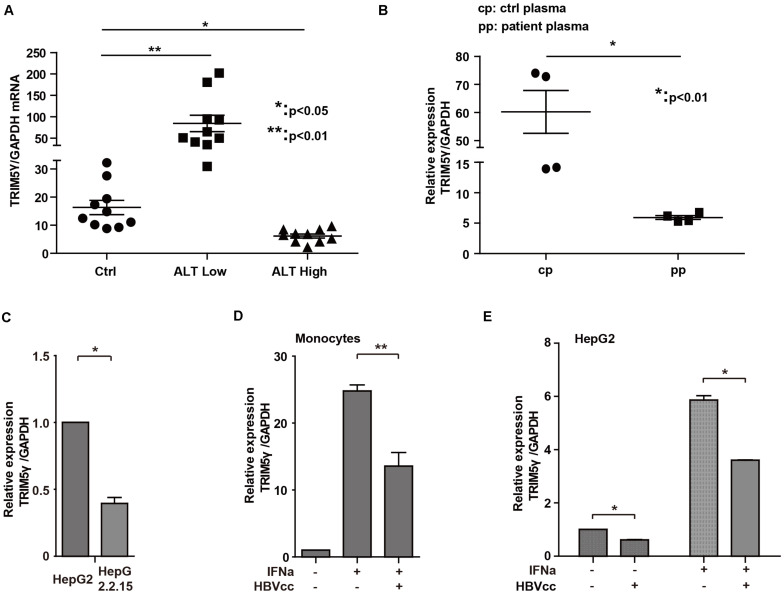
HBV inhibits TRIM5γ expression. **(A)** Total RNA was extracted from PBMCs isolated from 10 healthy individuals and 20 HBV patients. The expression level of TRIM5γ was analyzed using quantitative real-time PCR (qRT-PCR). **(B)** HepG2 cells were treated with plasma from 4 healthy individuals or 4 HBV patients, as indicated, and RNA was extracted and qRT-PCR was conducted to determine the TRIM5γ expression. **(C)** TRIM5γ expression in HepG2 or HepG2.2.15 cells was determined by q-PCR. **(D,E)** Monocytes or HepG2 cells were treated with IFNα or together with supernatant from HepG2.2.15 cells (HBVcc), as indicated. RNA was extracted and qRT-PCR was conducted to determine the TRIM5γ expression. *p < 0.05; **p < 0.01.

In our previous study, high TRIM5γ induction was noted, which indicated a better therapeutic effect in IFN-a treatment of HBV patients. Here, we investigated the expression of TRIM5γ in HBV patients. Interestingly, TRIM5γ expression demonstrated a much different pattern in the ALT low and high groups. It was highly expressed in the ALT low group, but less expressed in the ALT high group (Figure 5A), which indicated the different immune responses during different periods of hepatitis B virus infection. In addition, the plasma of HBV patients significantly inhibited the TRIM5γ expression in HepG2 cells (Figure 5B), which was consistent with our results. In fact, TRIM5γ expression was much lower in HepG2.2.15 cells (Figure 5C), which is a cell line with the HBV genome inserted. Finally, IFN-induced TRIM5γ expression was significantly inhibited when cells (Monocytes or HepG2) were cultured with the supernatant from HepG2.2.15 cells (Figures 5D,E). Cumulatively, these data suggest that the TRIM5γ expression was inhibited by HBV.

## Discussion

As a broad-spectrum antiviral drug, type-I IFN therapy remains an important approach for the treatment of HBV-infected patients. However, due to its severe side-effects and immune tolerance, its clinical application is restricted. Thus, understanding the mechanisms involved in the induction of anti-virus genes and the interaction of virus with the host protein is important as well as urgent. In this study, based on our previous research findings about TRIM5γ, we confirmed the TRIM5γ-HBx-DDB1 interactions in the HBV-infected PHH cells, and we found that IFN-induced TRIM5γ expression was STAT3-, and not STAT1-dependent, as well as that STAT3 directly binds to the promoter region of the TRIM5γ genome. We also found that the zinc-binding site H123 plays an indispensable role in the HBx-TRIM5γ BBOX interaction. Interestingly, a small peptide based on the BBOX domain was found to inhibit HBV replication.

In response to virus infection, the host defense system responds immediately, and cytokines are induced that bind to the signal transduction receptors for the inhibition of virus infection and replication. Moreover, types I and II cytokine-receptor superfamily, as one large subgroup, include receptors that bind to IFNs, several ILs, and colony-stimulating factors (McNab et al., 2015). All these cytokines employ the same mechanism of signal transduction, that is, the JAK–STAT pathway. JAKs are activated by cytokine binding, and, in turn, by phosphorylated cytokine receptors (Villarino et al., 2017). The members of the STAT family (STAT1, STAT2, STAT3, STAT4, STAT5A, STAT5B, and STAT6) become tyrosine-phosphorylated, which allows their dimerization, translocation to the nucleus, and binding to the promoters of the target genes (Yu and Jove, 2004). Recent evidence suggests the crucial role of STAT family proteins, especially STAT3, in inflammation and immunity (Okada et al., 2015; Xu et al., 2016; Song et al., 2019; Prado et al., 2020). In response to type-I IFN stimulation, STAT1, STAT2, and IRF9 first formed a Trimer complex that translocated to the nucleus for the promotion of downstream gene transcription. Consistently, we reported that TRIM14 induction was STAT1-, but not STAT3-dependent (Tan et al., 2018c). However, in this study, we found that STAT3, but not STAT1, played a critical role in IFN-induced TRIM5γ expression as a result of the direct binding of TRIM5γ to the promoter region and the resultant promotion of degradation of HBx, which confirmed the important role of STAT3 in the immunological defense after virus infection.

Small-molecule drugs (typically < 1000 Da) are believed to be the gold standard for drug development (Crook et al., 2020). In our previous study, we found that a 40-amino acid peptide was sufficient to promote the degradation of HBx protein. Accordingly, we further assessed whether a much smaller peptide would play the same role considering that H123 in the BBox domain was found to be critical in the interaction of HBx and BBox. We therefore proposed that the several amino acids around the binding site may be sufficient to promote the degradation of HBx; thus, 4 aa (BZ1) and 7 aa (BZ2) around the zinc binding site were picked and overexpressed in HepG2 cells together with HBx plasmids. Interestingly, we found that the HBx expression was inhibited by BZ2, but not BZ1, which was consistent with our conclusion that H123 was the binding site between HBx and BBox. In addition, we further confirmed that two 4-amino acid peptides were individually sufficient to promote HBx degradation in 293T and HepG2 cells, albeit for an unknown reason, the 4-amino acid peptide could not inhibit HBV replication (data not shown). Nevertheless, BZ2, which is a 7-amino acid peptide possessed the potentiality for further small peptide drug development.

**FIGURE 6:**
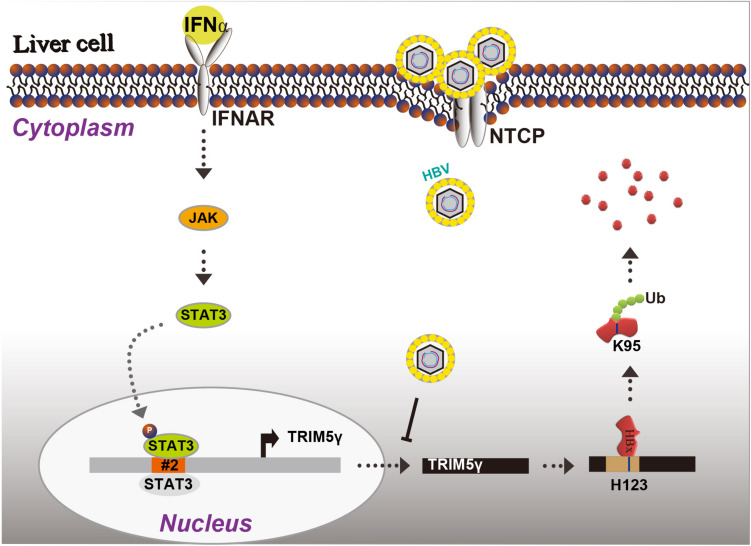
A working model for STAT3-dependent TRIM5γ in mediating the crosstalk between type-I IFN and HBV infection.

Finally, we propose a working model for STAT3-dependent TRIM5γ in mediating the crosstalk between type I IFN and HBV infection based on these findings (Figure 6). In conclusion, we found that the type I IFNs activated STAT3 to promote TRIM5γ gene expression. The H123 Zinc binding site in the BBox domain was responsible for the interaction and degradation of HBx, and BZ2, which is a 7aa peptide, might be a potential candidate for drug development, in addition, TRIM5γ expression is inhibited by HBV.

## Data Availability Statement

The original contributions presented in the study are included in the article/Supplementary Material, further inquiries can be directed to the corresponding author.

## Ethics Statement

The studies involving human participants were reviewed and approved by the First Hospital of Jilin University. Written informed consent for participation was not required for this study in accordance with the national legislation and the institutional requirements.

## Author Contributions

HS performed the experiments and wrote the paper. FX and XP performed the experiments. QX collected the patient’s samples. QW, BL, XL, and XF provided technical assistance and facility. GT planned, designed, and revised the manuscript. All authors contributed to the article and approved the submitted version.

## Conflict of Interest

The authors declare that the research was conducted in the absence of any commercial or financial relationships that could be construed as a potential conflict of interest.

## Supplementary Material

Supplementary Figure 1 Whole-Cell lysates of PHH cells were subjected to DDB1 pull down assay, and immunoblotted with TRIM5γ and DDB1 antibodies.

Supplementary Figure 2 HepG2 cells were transfected with BBox, B1, B2, or B3 Plasmids alone or left untransfected, 36 h later, cells were collected and subjected immunoblotting using GST or GAPDH antibodies as indicated.

Supplementary Table 1 Chronically HBV infected patients and healthy control.

>Supplementary Table 2 Primers related to q-PCR or CRISPR/Cas9 knockout.
