# Supplementary figures and images for: STAT3-Dependent Gene TRIM5γ Interacts With HBx Through a Zinc Binding Site on the BBox Domain
Source: Front Microbiol. 2021 Jul 2;12:663534. doi: 10.3389/fmicb.2021.663534 (PMC8283784; doi:10.3389/fmicb.2021.663534)

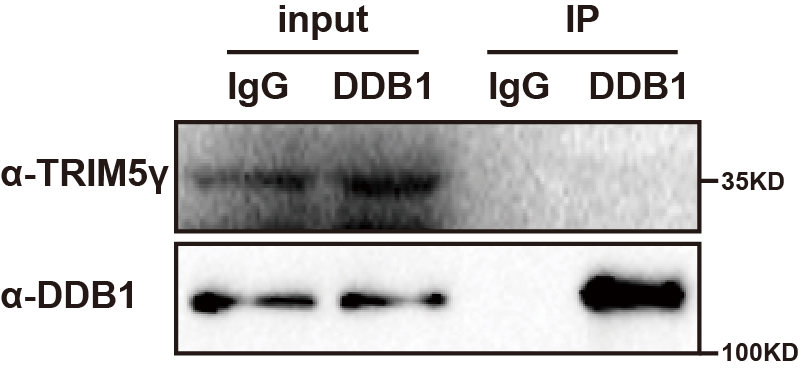

Supplement: Supplementary Figure 1 — Whole-Cell lysates of PHH cells were subjected to DDB1 pull down assay, and immunoblotted with TRIM5γ and DDB1 antibodies. [file Image_1.JPEG]

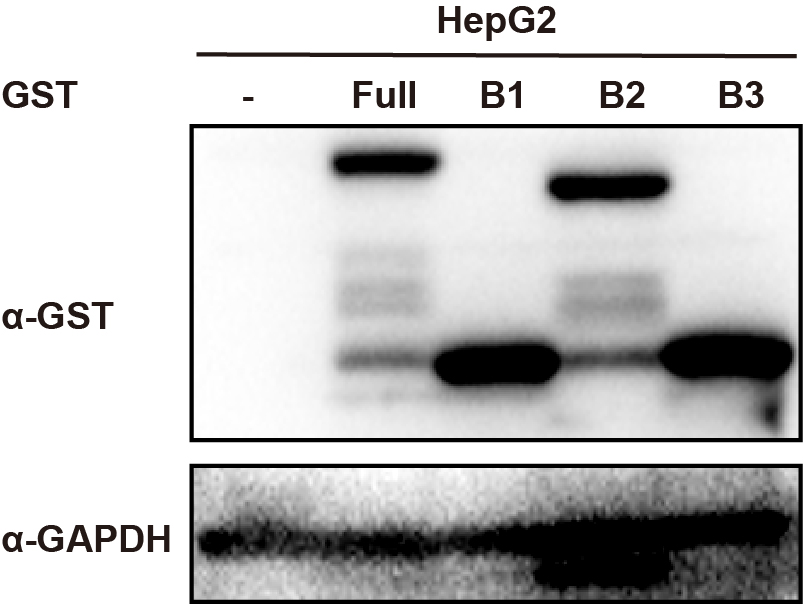

Supplement: Supplementary Figure 2 — HepG2 cells were transfected with BBox, B1, B2, or B3 Plasmids alone or left untransfected, 36 h later, cells were collected and subjected immunoblotting using GST or GAPDH antibodies as indicated. [file Image_2.JPEG]
