# Supplementary material for: STAT3-Dependent Gene TRIM5γ Interacts With HBx Through a Zinc Binding Site on the BBox Domain
Source: Front Microbiol. 2021 Jul 2;12:663534. doi: 10.3389/fmicb.2021.663534 (PMC8283784; doi:10.3389/fmicb.2021.663534)
Supplement: Supplementary Table 1 — Chronically HBV infected patients and healthy control. [file Table_1.DOCX]

Supplementary Materials:

**Table S1.** Chronically HBV infected patients and healthy control

| **ALT High**  **/Low** | **HBV patients** | | | | **Healthy Control** | | |
| --- | --- | --- | --- | --- | --- | --- | --- |
|  | Sex | Age | HBVDNA  (10^6^copies/ml) | ALT |  | Sex | Age |
| H1 | M | 25 | 116 | 907 | C1 | M | 35 |
| H2 | M | 27 | 165 | 1245 | C2 | F | 46 |
| H3 | F | 31 | 47.4 | 694 | C3 | F | 33 |
| H4 | M | 27 | 0.0874 | 1972 | C4 | M | 28 |
| H5 | F | 38 | 0.0369 | 441 | C5 | F | 36 |
| H6 | M | 40 | 5.77 | 499 | C6 | M | 32 |
| H7 | M | 48 | 0.27 | 706 | C7 | M | 39 |
| H8 | M | 27 | 113 | 1105 | C8 | F | 58 |
| H9 | F | 36 | 2.32 | 930 | C9 | M | 55 |
| H10 | M | 25 | 7.55 | 1316 | C10 | F | 58 |
| L1 | M | 42 | 9.07 | 138 |  |  |  |
| L2 | F | 47 | 3.46 | 60 |  |  |  |
| L3 | M | 36 | 4.54 | 66 |  |  |  |
| L4 | F | 50 | 1.54 | 85 |  |  |  |
| L5 | F | 29 | 5.58 | 230 |  |  |  |
| L6 | F | 28 | 0.047 | 63 |  |  |  |
| L7 | F | 47 | 346 | 60 |  |  |  |
| L8 | F | 50 | 154 | 85 |  |  |  |
| L9 | F | 56 | 21.6 | 126.7 |  |  |  |
| L10 | M | 35 | 102 | 112.8 |  |  |  |

| **Gene** | **Forward** | **Reverse** |
| --- | --- | --- |
| GAPDH | CGGATTTGGTCGTATTGGG | TCTCGCTCCTGGAAGATGG |
| HBVDNA | GAGTGTGGATTCGCACTCC | GAGGCGAGGGAGTTCTTCT |
| pgRNA | TCTTGCCTTACTTTTGGAAG | AGTTCTTCTTCTAGGGGACC |
| TRIM5γ | TATCATAAGCCACCCTGCGG | TGTGTGTCTTGGAAGGAGAATCA |
| TRIM5 sgRNA1F | CACCgTGGTAACTGATCCGGCACAC | AAACGTGTGCCGGATCAGTTACCAc |
| TRIM5 sgRNA2F | CACCgCGATTAGGCCGTATGTTCTC | AAACGAGAACATACGGCCTAATCGc |

**Table S2.** Primers related to q-PCR or CRISPR/Cas9 knockout
